# The effect of excluding juveniles on apparent adult olive baboons (*Papio anubis*) social networks

**DOI:** 10.1371/journal.pone.0173146

**Published:** 2017-03-21

**Authors:** Piotr Fedurek, Julia Lehmann

**Affiliations:** Department of Life Sciences, University of Roehampton, London, United Kingdom; University of Tasmania, AUSTRALIA

## Abstract

In recent years there has been much interest in investigating the social structure of group living animals using social network analysis. Many studies so far have focused on the social networks of adults, often excluding younger, immature group members. This potentially may lead to a biased view of group social structure as multiple recent studies have shown that younger group members can significantly contribute to group structure. As proof of the concept, we address this issue by investigating social network structure with and without juveniles in wild olive baboons (*Papio anubis*) at Gashaka Gumti National Park, Nigeria. Two social networks including all independently moving individuals (i.e., excluding dependent juveniles) were created based on aggressive and grooming behaviour. We used knockout simulations based on the random removal of individuals from the network in order to investigate to what extent the exclusion of juveniles affects the resulting network structure and our interpretation of age-sex specific social roles. We found that juvenile social patterns differed from those of adults and that the exclusion of juveniles from the network significantly altered the resulting overall network structure. Moreover, the removal of juveniles from the network affected individuals in specific age-sex classes differently: for example, including juveniles in the grooming network increased network centrality of adult females while decreasing centrality of adult males. These results suggest that excluding juveniles from the analysis may not only result in a distorted picture of the overall social structure but also may mask some of the social roles of individuals belonging to different age-sex classes.

## Introduction

The prevailing view on animal social structure appears to be that adults are the key players in organising the social structure of the group. Indeed, studies conducted on long-lived mammals, such as elephants and primates, suggest that adults play a central role in maintaining social stability [1–4]. The influence that adults have on the social cohesion of the group has been attributed to their foraging experience (e.g., knowledge of feedings sites) and established social relationships in the group as well as their dominance status ([1,5–7], but see [8]). However, in some mammals, juveniles have also been shown to influence social cohesion of the group; e.g. by being the main recipient of affiliative interactions juvenile yellow-bellied marmots (*Marmota flaviventris*) increase the cohesion of the affiliative network [9]. Similarly, juveniles were reported to have a strong effect on social network structure of African lions (*Panthera leo*) [10] and killer whales (*Orcinus orca*) [11]. These studies suggest that including juveniles in the analyses can have a significant impact on the interpretation of social structure.

It has recently been shown that incomplete networks can provide robust results and conclusions as long as there is no bias toward who gets included [12]. However, excluding entire age (or age-sex) classes from the analysis may affect the conclusions in certain contexts, depending on the research questions being asked. For example, when network analysis is used in order to quantify parasite or disease transmission, a representative sample of all individuals, including all age classes, will be crucial for our understanding of the underlying process as juveniles may play a key role in transmitting diseases. This could be especially important if juveniles affect the centrality of specific age-sex classes differently in which case both, the number of juveniles and the sex ratio of adults are expected to interact in terms of, for instance, their effects on disease transmission. While some studies do include juveniles (e.g., [13–14]), others do not (e.g., [15–17]) and the consequences of including or excluding juveniles from analyses are rarely discussed. A similar argument could be made for studies on the diffusion of information through social networks.

Here, we aim to test the hypothesis that the inclusion/exclusion of juveniles can significantly affect the apparent social network structure and the interpretation of social roles, using a group of primates as our study species. Primates are known to have highly differentiated social relationships [18], however, most studies on their social network structure only include adults and subadults [19–22], while juveniles are often ignored, mainly due to logistic and technical rather than theoretical reasons. The slow physical development of primates results in a largely extended period of juvenility [23–24] and sexually immature individuals can account for a relatively large proportion of the group [25]. However, juveniles are often ignored and the effect of this age class on the topography of social structure remains poorly understood. Moreover, numerous studies of primates have found that juveniles are frequently involved in social interactions with other, often unrelated group members [26–30]; suggesting that they do have the potential to markedly affect the overall social structure and social dynamics of the group.

In our study, we use olive baboons (*Papio anubis*) as a model species to assess how social networks change with the inclusion of juveniles and to what extent this affects the conclusions about the social roles of the other age classes. Baboons are a suitable model for such analyses, as they, form multi-male, multi-female groups, where females remain in the natal group and form strong social relationships with their female kin while males disperse into a new troop at maturity [31]. They also have been shown to have highly differentiated social relationships [21,32–34], which can have profound fitness consequences [32–35], for example by enhancing an individual’s longevity [36] and offspring survival [37–38]. An individual’s social role is influenced by its age, sex, rank and kin relationships [39–40]. It has been shown that in baboons even immature individuals can have differentiated social relationships with other individuals, which are structured not only by kinship and maternal rank (e.g., yellow baboons *Papio cynocephalus* [41–42]) but also by age and sex (e.g., Chacma baboons (*Papio ursinus*) and olive baboons [27–28,43]). Thus, it is likely that immature individuals contribute independently to the overall social structure of the group, although this has rarely been investigated systematically.

Here, we use social network analysis (SNA) in combination with knock-out simulations to assess (i) to what extent juvenile and adult olive baboons (*Papio anubis*) differ in their social network measures (ii) how the inclusion/exclusion of juveniles affects the apparent social network structure and (iii) whether the conclusions drawn about the relative social roles of different age-sex classes changes depending on whether or not juveniles are included into the social networks. We use two different types of social networks, namely grooming and aggression networks, as these have been shown to measure different aspects of baboon sociality [44] and social integration in these networks can have important survival and fitness consequences [45]. We predict that in line with previous studies [42] juveniles and adults differ in the extent to which they are connected in their social network [42,46]. Furthermore, we expect that including juveniles into the social network will have a 'diluting' effect on the overall topology of the grooming and aggression networks because studies on baboons have shown that juveniles groom primarily with their mothers (e.g, chacma baboons [27]) and, although they interact antagonistically with adults (e.g., yellow [41,47] and olive baboons [43]), they are thought to not yet be fully integrated in the aggression network. Finally, because in olive baboons (as well as in many other multi-male, multi-female Old World monkeys exhibiting similar social system as olive baboons, such as chacma baboons [42] and vervet monkeys *(Chlorocebus pygerythrus* [48]) adult males and females have been found to differ in their interaction patterns with juveniles [43,49], we predict that the exclusion of juveniles will have sex-specific effects on the network metrics of adults, depending on which behaviours are used to create the social networks. For example, given the fact that in olive baboons adult females interact antagonistically with juveniles more often than males do [49], an obvious (but not previously tested) expectation would be that the social network position of adult males will be affected to a lesser extent by the removal of juveniles than those of adult females.

## Methods

### Study subjects

This study was conducted in Gashaka Gumti National Park (6°55′N 11°13′E), Nigeria, on a well habituated troop of olive baboons. The troop’s home range is characterised by a mixture of various habitats types, including lowland forest, Southern Guinea savannah woodland, gallery forest and grassland [50–51]. Data collection was conducted over a three-month period, between March and June 2013 (dry season). During the study period, group size varied between 28 and 30 individuals, with four adult males (exhibiting fully developed secondary sexual characteristics, aged 8+ yrs), eight adult females (who had reproduced, approximate age: 5+ yrs), four subadult females (who had started cycling but have not reproduced yet, aged 4-5 yrs), one natal subadult male (bigger than adult females with well-developed secondary sexual characteristics but had not started mating, aged 6-7 yrs), eight juvenile males (fully weaned, smaller than subadult males, without a mantle and shoulder hair; aged 2-6 yrs), two juvenile females (fully weaned but not yet cycling, aged 2-4 yrs) and 1-3 dependent infants (two infants were born during the study period). Age-sex classes were defined after Warren (2003) [52].

### Data collection

Data on social interactions were collected from 25 individuals, excluding the three dependent infants, one newly immigrated (and thus not yet habituated) adult male and one adult female, who was very shy of human presence and difficult to follow on a regular basis. All 25 study subjects were fully habituated and did not appear to be disturbed by human presence. Data were collected using focal animal sampling [53]. One-hour focal follows were conducted between 06:00 am and 03:00 pm by PF. Focal subjects were chosen pseudo-randomly, ensuring that individuals were observed roughly equally often and that observation times per individual were evenly distributed across the times of the observation day. A total of 204.58 hours of data were collected, with a mean observation time per individual of 8 hrs (SD = 0.52h, min 6.32h, max 10.14h). Each study subject was followed approximately 7 times (SD 1.18).

We recorded the following social behaviours: allogrooming (cleaning the fur and skin of a partner using fingernails or/and teeth) and agonistic interactions (physical aggression, such as bite, chase, hit, displacement and visual threats). For these social interactions we recorded the frequency, duration and identity of the partner. With regard to grooming, a new bout was recorded when the grooming partner changed, the direction of grooming changed, or when individuals interrupted grooming for more than 30s [54].

### Social network analysis

We constructed two social networks: one based on grooming behaviour and one based on aggressive behaviours. We chose these two behavioural categories as it has been shown that in some mammals aggression and grooming social networks play an important role in terms of survival (e.g., feral horses *Equus ferus* [55], Barbary macaques *Macaca sylvanus* [45,56]). Each network initially included all study subjects (*n* = 25). In the grooming network, ties represent time (seconds per hour) a given dyad was engaged in grooming. Because agonistic behaviours are often short and durations cannot be accurately measured, these networks were based on dyadic interaction rates (number of agonistic interactions observed between two individuals per hour). Both networks were directional (asymmetric) and weighted.

First, in order to assess if juveniles and adults differ in their overall level of social integration, number of social partners and the strength of social relationships (aim 1), we compared the following frequently used network metrics between juveniles and adults: degree, in-/out-degree, in-/out-strength, betweenness centrality and individual clustering coefficient. *Degree* (derived from symmetric matrices) indicates the number of social partners with whom an individual is involved in a particular activity (e.g., gooming). *In-degree* indicates the number of social partners that initiate the social interaction to an individual while *out-degree* shows the number of social partners with whom an individual initiates interactions. *In-strength* measures the overall strength (interaction frequencies) of social interactions received by an individual (i.e., the sum of the weights of all in-coming ties) while *out-strength* indicates the cumulative strength of initiated interactions (i.e., the sum of weights of all out-coming ties). *Betweenness centrality* indicates how often an individual lies on the shortest path between any other dyad [57] and has important implications for network stability [58] and disease transmission [59]. *Individual* c*lustering coefficient* measures the degree to which the interaction partners of an individual are interacting among themselves and is calculated as the proportion of the existing ties to all possible ties between the individual's partners [60–61]. In other words, it shows the extent to which social interactions occur primarily within sub-groups. Second, to assess the apparent effect the exclusion of juveniles has on overall network structure, we calculated three commonly used global network parameters [12,62–63]: *density*, *network degree centralisation* and *mean clustering coefficient*. Global network measures, as opposed to individual network measures, provide a description of the network as a whole and are not attributed to particular individuals [64]. These network parameters have been suggested as different indices of overall network cohesion and measure important overall network properties [11,15,44,54,63]. *Density* measures the number of existing ties in relation to the number of possible ties in a network [65]. *Network degree centralisation* measures the extent to which social interactions are centred on particular individuals and provides a good estimate of how evenly social interactions are distributed across the network [65]. Here we used a standardised measure of this metric (ranging from 0 to 1), where values close to zero indicate that all individuals are similarly involved in social interactions while values closer to 1 indicate that a small number of individuals are involved in a disproportionally high number of interactions. *Mean clustering coefficient* indicates to what extent, on average, individuals connected to one individual are also connected themselves [65–66].

The importance of using weighted networks in animal studies has been emphasised on numerous occasions [67–68]. Although we used weighted networks whenever possible, some measures, such as (in/out) degree, network degree centralisation and density are (by default) based on unweighted networks (i.e. indicating the presence or absence of a tie, but ignoring the strength of ties [65]) and were calculated using igraph package [69] for R [70]. All other individual and global measures (i.e. strength, individual and mean clustering coefficient, betweenness) were calculated as weighted measures, using the tnet package [71] for R. While calculating the network metrics we used the alpha function available in tnet which specifies the weight given to the presence of a tie versus its strength. To calculate tie strength, the alpha parameter was set on 1 (ignoring the number of ties), while for all other measures the number of ties and the strength of the relationships were given equal weight in the calculation of network parameters (alpha = 0.5; [71]). For the calculation of clustering coefficients, networks were symmetrised (using ‘symmetrise’ function available in tnet which adds up weights from both directions in order to symmetrise the matrix) as this metric is currently not well defined for asymmetric, directed networks.

### Knock-out simulations

In order to determine what effect the removal of juveniles has an on the apparent structure of the grooming and aggression network (aim 2), we compared network metrics between networks with and without juveniles. However, because network size and density can influence network metrics [54], networks with and without juveniles cannot be compared directly. Instead, we use simulated knockout simulations, removing individuals from the network in either a targeted or a random fashion, and subsequently compare the network parameters of these simulated networks. This allows us to compare networks of the same size. We used three different types of knockout simulations: removal of juveniles from the network, removal of adults from the network and removal of random individuals from the network. Removing all 10 juveniles from the network created a network containing only adults and subadults (thus, representing results from a study omitting juveniles); removing up to 10 adults from the network created networks of the same size to the juvenile-removal simulation, but containing a larger proportion of juveniles and subadults (thus, highlighting the social networks of younger group members). Removing random individuals from the network is a control condition for the reduction in network size *per se*. All removals were done in a stepwise fashion and network parameters were calculated at each step. The stepwise removal of individuals allowed us also to assess the direction of change and its linearity as well as to determine whether the slope gradients of change in network parameters resulting from the removals differed between adults and juveniles. For targeted removals, all possible combinations of removed individuals were used at each step, following the equation:

$$N\;combinations=\frac{n!}{r!(n-r)!}$$

where n is the total number of targeted individuals (here n = 10) and r is the number of removed individuals. For example, removing 4 out of 10 juveniles from the group results in 210 different combinations, all of which were simulated. For the random ‘control’ removals, individuals to be removed were chosen randomly (i.e., irrespectively of their age or sex) from the group and the procedure was repeated 500 times [except for the first two removals where, in order to avoid pseudoreplication, we used the permutation-derived knockout procedure as during the targeted removals (as the number of possible combinations for the first two steps is less than 500)].

In order to determine how juveniles affect network metrics of individuals belonging to different age-sex classes (aim 3) rather than network metrics overall, we performed simulated removals of juveniles using the same step-wise procedure described above with 50 iterations at each step. At every removal step we calculated separate averages of the network parameters for adult males, adult females and subadult females. Due to the small number of subadult males (*n* = 1), we excluded this age-sex class from the analysis. We only used two of our individual network measures, namely mean betweenness centrality (indicating a mean value of betweenness centrality of all individuals belonging to a given age-sex class) and mean clustering coefficient, as both degree and strength measures are expected to change systematically with overall network size and are thus of little interest in this context. We also assessed whether the relative centrality positions of individuals belonging to different age sex classes remained the same for networks with and without juveniles. In order to do so, each individual was assigned a rank corresponding to their betweenness values. We then compared these rank positions between networks containing only adults and those containing juveniles as well to assess if the inclusion of juveniles into social networks can alter the conclusion about age/sex specific social roles.

Finally, because network metrics, such as clustering coefficients, have been shown to be sensitive to changes in network density [54,62], we also controlled for changes in density (see **S1 Text**).

### Statistical analysis

First, we tested to what extent individual network metrics, such as degree, strength, betweenness and clustering are correlated with each other using Kendall rank correlation. In order to determine whether juveniles and adults differed in terms of their age-class network measures (aim 1), we generated a simulated distribution of mean network metrics of adults and juveniles by performing a node permutation within each of the two age-sex classes. Because a random node permutation within a given age-class will always produce the same mean value of a given network metric, we performed a selective node permutation without replacement by drawing only five individuals from a given age-class during each iteration. Given that there are 252 different combinations in selecting five out of ten juveniles/adults, we considered all these possible combinations in deriving the metric distributions of the two age-sex classes. We then compared the simulated distributions of network metrics of adults with the corresponding metric distributions of juveniles using Wilcoxon sum-rank test (as the Shapiro-Wilk test showed that most of the simulated distributions were highly non-normal).

Second, we determined whether the removal of individuals from the social networks significantly changed the global network metrics (2^nd^ aim). Levene's test showed that variances between groups were not homogenous; thus, we used a Kruskal-Wallis (K-W) test with the number of removed nodes as the independent variable and respective network metrics as dependent variable. We conducted separate K-W tests for juveniles, adults and random removals to assess if the metrics changed following the removals. In addition, to assess if there were significant differences between the network metrics after the removal of adults compared to those following the removal of juveniles, we used Scheirer-Ray-Hare test (SRH; a factorial non-parametric test which uses Chi-square procedures in order to derive p values [72]) with removal category (2 levels: adults and juveniles) and the number of removed individuals (11 levels, i.e., following 0-10 removals) as independent variables and the respective network metric as dependent variables. We also used Mann Whitney U tests for a post-hoc analysis, comparing network metrics between the two age-classes.

Third, to assess if the removal of juveniles particularly affected a specific age-sex class (3^rd^ aim), we used pairwise comparisons of the slope parameters of the changes in network metrics following the stepwise removal of juveniles using regression analysis. This allowed us to assess if network metrics change systematically with the removal of juveniles and if this change differed between the different age-sex classes used here. In order to minimise the type 1 error resulting from multiple permutations (N = 50) used in producing each slope, slopes were derived from the mean metric values of individuals belonging to a given age-sex class after the stepwise removal of juveniles from a network. Finally, using independent t-test, we also compared bootstrapped (N = 1000) metric scores between age-sex classes before and after the removal of juveniles. This allowed us to determine whether age-sex class differences in networks with and without juveniles were the same.

The simulations and statistical analyses were conducted in R using the following packages: bipartite [73], combinat [74] and picante [75]. The SRH test was run using R codes introduced by Dytham (2011) [76].

## Results

### Aim 1) Differences in social network metrics between juveniles and adults

**Fig 1:**
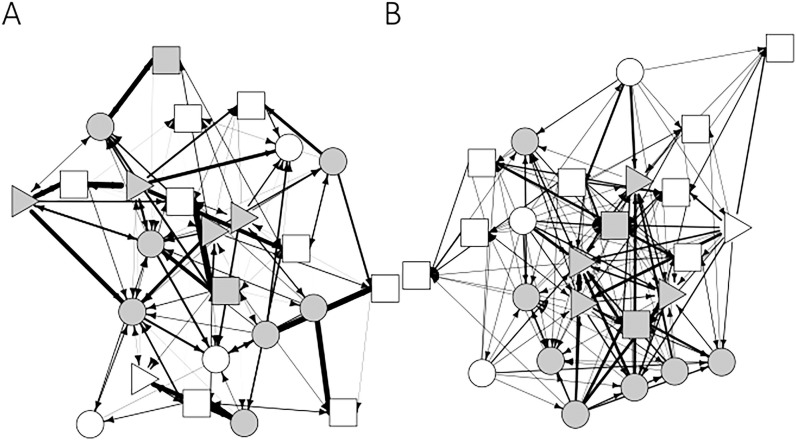
**Graphic representation of the grooming (A) and aggression network (B)**.

We recorded 319 grooming bouts and 272 agonistic interactions. Thus, social interactions were relatively rare in this group of baboons [44]. However, both networks are well connected and not too sparse (Fig 1). **S1 Table** (Supplementary Material) shows the percentage distribution of grooming and agonistic interactions among specific age sex classes, indicating that females participated on the majority of grooming and aggressive interactions.

Squares, triangles and circles represent juveniles, subadults and adults respectively. White nodes represent males, grey nodes represent females. Lines represent grooming (A) and aggressive (B) interactions between individuals. The line thickness indicates the proportion of time spent grooming or the hourly rate of agonistic interactions. The graphs are laid out using ‘spring embedding’ procedure, which places individuals in such a way that those with the smallest distance to one another are closest to each other in the graph.

Although some network metrics were significantly correlated with each other (e.g. degree and strength; **S2 Table,** Supplementary Material), we maintained all measures in the analysis because none of the metrics was correlated with all other measures and correlations differed between networks (**S2 Table)**, thus not leading to surreptitious information. Overall, the difference between juveniles and adults in the network metrics were highly pronounced in both social networks as most metrics (11 out of 12) differed significantly between the two age-classes (Table 1). In the grooming network juveniles were significantly more central compared to adults (as indicated by betweenness centrality) and groomed a significantly larger number of individuals (out-degree) although less frequently (out-strength) but received grooming from a significantly smaller number of group members (in-degree) compared to adults (Table 1; **Fig A in S2 Text**, **Fig B in S2 Text**, **Fig D in S2 Text** and **Fig E in S2 Text**). In the agonistic network, adults were found to be more central (betweenness centrality) and initiated aggression significantly more frequently (out-strength) and to a significantly higher number of individuals (out-degree) compared to juveniles (Table 1, **Fig H in S2 Text**, **Fig J in S2 Text** and **Fig K in S2 Text**). On the other hand, juveniles received aggression from a significantly higher number of individuals and more frequently (in-strength) than did adults (Table 1; **Fig G in S2 Text** and **Fig I in S2 Text**.

**Table 1:** Mean values of the individual network metrics of juveniles and adults in the grooming and aggression network.

Network type	Age-class	In degree	Out degree	In strength	Out strength	Betweenness	Clustering
**Grooming**	Juveniles	**4.9 (2-9)**	**4.8 (1-15)**	124.4 (2.2-20)	**69 (1.9-314)**	**42.4 (0-118)**	**0.4 (0.14-0.76)**
	Adults	**6.0 (2-12)**	**3.9 (0-7)**	121.4 (2.3-24.9)	**104 (0-227)**	**32.1 (0-101)**	**0.43 (0.29-0.66)**
**Agonistic**	Juveniles	**6.5 (1-16)**	**4.4 (0-12)**	**0.71 (0.25-1.95)**	**0.51 (0-1.32)**	**18.6 (0-94)**	**0.52 (0.41-0.69)**
	Adults	**4.1 (0-9)**	**6.9 (1-12)**	**0.4 (0-093)**	**0.83 (0.12-1.9)**	**22.1 (0-82)**	**0.56 (0.41-0.71)**

The observed range values of the metrics are in brackets. Significant results of the resampled replicates (N = 252) are in bold.

### Aim 2) The effect of juvenile exclusion on apparent global network structure

As expected, the majority of network metrics (11 out of 18) of both networks changed significantly following the removal of individuals (Table 2). Of the seven non-significant values five occurred following random removals, suggesting that random removals overall are less likely to affect network parameters than targeted removals. Specifically, density and clustering were not affected by random removals in either of our two networks (Table 2).

**Table 2:** Kruskal-Wallis test results showing the effects of the stepwise removal of 1-10 individuals (adults, juveniles or random individuals) on global network metrics for two different social networks.

Network type	Metric	Adults removed	Juveniles removed	Random removal
Grooming	Density	• χ^2^ = 1.80 • P = 0.99	• χ^2^ = **31.79** • **P<0.001**	• χ^2^ = 8.83 • P = 0.55
	Clustering_coef_	• χ^2^ = **212.60** • **P<0.001**	• χ^2^ = **62.11** • **P<0.001**	• χ^2^ = 18.04 • P = 0.054
	Centralisation	• χ^2^ = **69.05** • **P<0.001**	• χ^2^ = **413.86** • **P<0.001**	• χ^2^ = **71.24** • **P<0.001**
Aggression	Density	• χ^2^ = **392.88** • **P<0.001**	• χ^2^ = **499.84** • **P<0.001**	• χ^2^ = 3.43 • P = 0.96
	Clustering_coef_	• χ^2^ = **288.75** • **P<0.001**	• χ^2^ = **378.92** • **P<0.001**	• χ^2^ = 11.91 • P = 0.29
	Centralisation	• χ^2^ = **52.61** • **P<0.001**	• χ^2^ = 16.44 • P = 0.09	• χ^2^ = 15.09 • P = 0.13

Significant results are in bold; all DFs = 10

#### Grooming network

Network parameters changed significantly following the removal of all targeted individuals (Table 3: all main effects of removals significant). In addition, we found that the removal of adults generally produced effects that were significantly different from those following the removal of juveniles (Table 3; all main effects removal category significant and all interactions significant).

**Table 3:** Results of the Schreier-Ray-Hare test to assess if the removal of adults and juveniles affected network metrics differently.

		Main effect:		Interaction:
Network	Metric	‘Removal’	Removal category’	Removal’ x ‘category’
Grooming	Density	**• H = 22.20** **• P<0.001**	**• H = 24.61** **• P<0.001**	**• H = 7.50** **• P = 0.006**
	Clustering_coef_	**• H = 11.2** **• P<0.001**	**• H = 1006.3** **• P<0.001**	**• H = 109.6** **• P<0.001**
	Centralisation	**• H = 40.15** **• P<0.001**	**• H = 1036.89** **• P<0.001**	**• H = 174.66** **• P<0.001**
Aggression	Density	**• H = 807.72** **• P<0.001**	**• H = 53.61** **• P<0.001**	**•** H = 2.46 **•** P = 0.12
	Clustering_coef_	**• H = 642.43** **• P<0.001**	**• H = 49.89** **• P<0.001**	**•** H = 0.22 **•** P = 0.64
	Centralisation	**• H = 36.25** **• P<0.001**	**• H = 287.39** **• P<0.001**	**• H = 7.83** **• P = 0.005**

Significant results are in bold; Removals = stepwise removals from 1 = 10; removal category = juvenile or adult removal. Df = 2047

**Fig 2:**
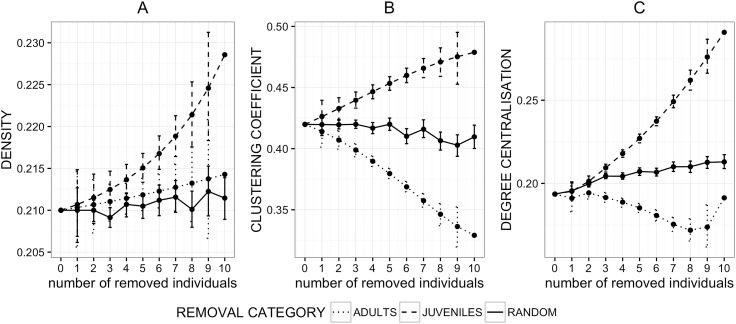
**Effects of random (solid line) and targeted (dashed and dotted line) removal of individuals from the grooming network on network density (A), clustering coefficient (B) and network degree centralisation (C)**.

Network density_Gr_, which did not change significantly following the removal of adults or random individuals, increased significantly when juveniles were removed (Table 2, Fig 2A). Similarly, clustering_Gr_ coefficient was not significantly affected by the random removal of individuals (Table 2), while targeted removal of adults or juveniles had opposite effects on clustering (except for the first step; see **S4 Table**): clustering_Gr_ coefficient decreased following the systematic removal of adults while it increased following the removal of juveniles (Fig 2B). Finally, network degree centralisation_Gr_ was also affected by all removals, with adults and juveniles having again opposite effects: the removal of juveniles increased network degree centralisation_Gr_, while the removal of adults decreased it (Fig 2C).

Each point represents a mean value of the network metrics following simulated, permutation-based removals of selected (within respective age classes) individuals. Error bars represent standard errors.

#### Aggression network

Although targeted removals had a significant effect on all aggression network metrics (Table 3, all main effects of removals significant), these effects were more uniform compared to grooming network, with adults and juveniles removals often producing similar effects (Table 3, two non-significant interactions).

**Fig 3:**
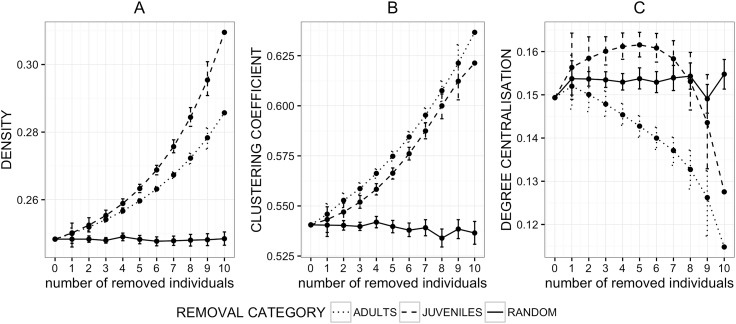
**Effects of random (solid line) and targeted (dashed and dotted line) removal of individuals from the aggression network on network density (A), clustering coefficient (B) and network degree centralisation (C)**.

Like in the grooming network, network density_Ag_ was not affected by random removals, but increased significantly following the removal of adults or juveniles, with a significantly stronger increase following the removal of juveniles (Fig 3A). Similarly, the mean clustering_Ag_ coefficient changed little following random removals (Table 2) but increased considerably following the removal of juveniles and adults, suggesting that both adults and juveniles significantly reduced clustering in the aggression network. Interestingly, following targeted removals (adults or juveniles) network metrics changed in a similar pattern in terms of the direction and linearity of the change. However, the effects differed in magnitude, with the removal of adults leading to a greater increase in the mean metric than the removal of juveniles (Fig 3B). The fact that random removals had no marked effect on network clustering_Ag_ compared to targeted removals suggests that subadult females, which were not included in knockout simulations, had an opposing effect on network clustering to adults and juveniles. Network degree centralisation_Ag_ decreased following the removal of adults, while it initially increased following the removal of juveniles before decreasing markedly (Fig 3C).

Each point represents a mean value of the network metrics following simulated, permutation-based removals of selected (within respective age classes) individuals. Error bars represent standard errors. Some of the error bars are very narrow and cannot be seen.

Finally, networks with a higher proportion of juveniles were more similar to random networks than networks comprising of only adults and sub-adults **(S6 Table** and **S7 Table**), suggesting that it is mainly adults who maintain non-random network structure (see **S1 Text** for more details).

### Aim 3) The influence of juveniles on apparent social network positions of individuals belonging to different age-sex classes

We assessed whether the removal of juveniles from the social networks had different effects on the separate age-sex classes because we were interested in the extent to which the inclusion of juveniles would alter the interpretation of sex-specific social roles. Our results show that age-sex classes were affected differently by the removal of juveniles from the network.).

#### Grooming network

The removal of juveniles affected all age-sex classes differently, with significantly different slope parameters in all pairwise comparisons (Table 4).

**Table 4:** Results of the pairwise slope comparisons of all three age-sex classes for two different social networks and two network parameters.

**Grooming**		Subadult females	Adult females
Clustering coefficient	Adult females	*F* = 26.2 *P*<0.001	
	Adult males	*F* = 65.9 *P* = 0.001	*F* = 126.1 *P* = 0.009
Betweenness centrality	Adult females	*F* = 10.4 *P* = 0.004	
	Adult males	*F* = 162.4 *P*<0.001	*F* = 247.9 *P*<0.001
**Aggression**		Subadult females	Adult females
Clustering coefficient	Adult females	*F* = 5.6 *P*<0.001	
	Adult males	*F* = 23.0 *P*<0.001	*F* = 43.1 *P*<0.001
Betweenness	Adult females	*F* = 37.4 *P*<0.001	
	Adult males	*F* = 333.4 P<0.001	*F* = 161.4 *P*<0.001

Regression slopes were derived from the mean network metrics of the age-sex classes after a simulated removal (50 iterations) of 1-10 juveniles from the grooming network. All *DFs* = 1,8.

**Fig 4:**
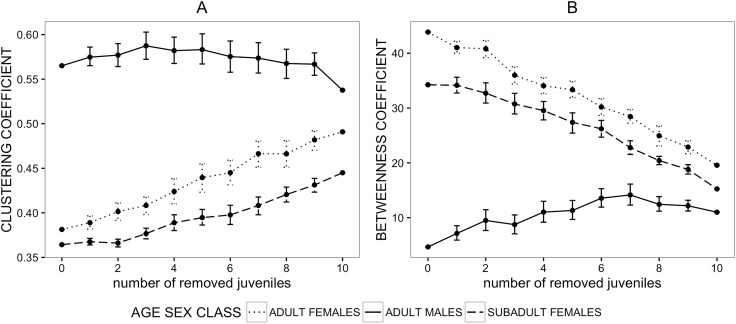
**Mean values of clustering coefficient (A) and betweenness centrality (B) after the removal of juveniles from the grooming network**.

The clustering_Gr_ coefficient of adult males was not strongly affected by the removal of juveniles; both adult and subadult females showed a linear increase in clustering_Gr_ coefficient following the removal of juveniles (Fig 4A). This suggests that the inclusion of juveniles into the network reduces the apparent existence of local grooming clusters in females, but not in males. Similarly, grooming betweenness_Gr_ centrality in adult males was affected to a lesser extent by juvenile removal than betweenness centrality of subadult or adult females, which decreased sharply following the exclusion of juveniles (Fig 4B).

Each point represents a mean value of the network metric of individuals belonging to a given age-sex class after a simulated removal (50 iterations) of randomly selected juveniles. Dotted, dashed and continuous lines represent network metric values of adult females, subadult females and adult males respectively after simulated removals of juveniles. Error bars represent standard errors. Some of the error bars are very narrow and cannot be seen.

However, although the exclusion of juveniles affected the network_Gr_ measures of age-sex classes differently, there was no significant change in the relative positions of age-sex classes to each other (Table 5).

**Table 5:** T-test results of the bootstrapped metric values of the three age-sex classes in the two social networks before (upper cells of the pairwise comparisons) and after (lower cells) the removal of juveniles.

**Grooming**		Subadult females	Adult females
Clustering coefficient	Adult females	t = 3.39 P<0.001	
		t = 21.47 P<0.001	
	Adult males	t = 22.31 P = 0.001	t = 20.86 P = 0.009
		t = 11.50 P<0.001	t = 8.80 P<0.001
Betweenness centrality	Adult females	t = 4.13 P = 0.004	
		t = 7.07 P<0.001	
	Adult males	t = 11.98 P<0.001	t = 17.55 P<0.001
		t = 5.33 P<0.001	t = 9.57 P<0.001
**Aggression**		Subadult females	Adult females
Clustering coefficient	Adult females	t = 6.36 P<0.001	
		t = 2.71 P = 0.007	
	Adult males	**t = 8.39 P<0.001**	t = 14.18 P<0.001
		**t = 0.04 P = 0.96**	t = 3.06 P = 0.002
Betweenness centrality	Adult females	t = 26 P<0.001	
		t = 12.10 P<0.001	
	Adult males	t = 40.25 P<0.001	**t = 22.39 P<0.001**
		t = 13.6 P<0.001	**t = 1.48 P = 0.14**

T-test scores where the differences between any two age-sex classes were significant before but not after the removals are in bold.

**Fig 5:**
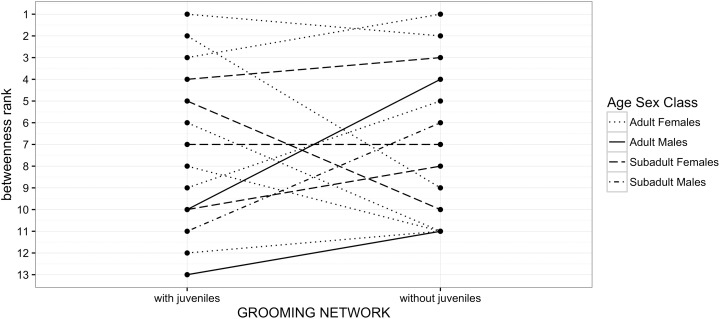
Changes in relative betweenness_Gr_ rank of different age-sex classes resulting from the removal of juveniles in the grooming network.

When comparing the five most central individuals (betweenness_Gr_ centrality) between networks with and without juveniles (Fig 5), we found some changes: two out of five individuals differed and the age-sex class composition of the top five individuals had changed from all females (adult and subadult) in the network with juveniles, to four females and one adult male in the network without juveniles (Fig 5).

The betweenness_Gr_ rank changes of the two bottom ranked males are depicted by the same solid line as their rank position is identical in the two networks (i.e., with and without juveniles).

#### Aggression network

**Fig 6:**
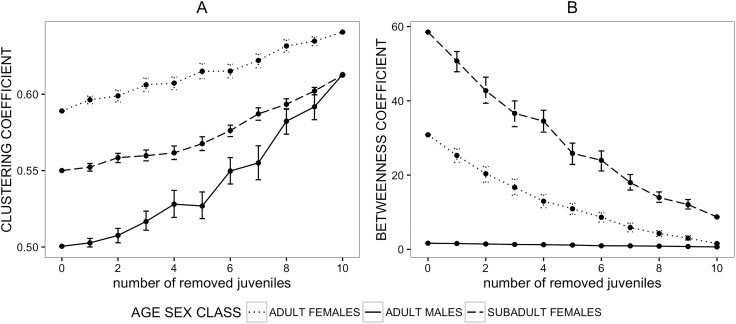
**Mean values of clustering coefficient (A) and betweenness centrality (B) after the removal of juveniles from the aggression network**.

The removal of juveniles affected age-sex classes differently with significantly different slope parameters in all pairwise comparison (Table 4). The removal of juveniles led to an increase of clustering_Ag_ values in the aggression network in all age-sex classes (Fig 6A). Interestingly, in the aggression network males’ clustering_Ag_ coefficient appeared to be most affected by the removal of juveniles, with values increasing more than those of adult and subadult females (Fig 6A). Male betweenness_Ag_ centrality was largely unaffected by the removal of juveniles (Fig 6B), whereas female values decreased when juveniles were removed, with subadult females being most strongly affected. We then tested if the changes after the removal of juveniles led to a different interpretation of age-sex class specific social network_Ag_ positions. Interestingly, when juveniles were excluded from the aggression network, there was no significant difference between subadult females and adult males in terms of clustering_Ag_ and no significant difference between adult males and females in betweenness_Ag_ centrality (Table 5). Only when juveniles were included into the network, did these age-sex classes differ significantly (Table 5), suggesting that inclusion of juveniles significantly affect apparent network structure and can alter the apparent age-sex differences in sociality.

Each point represents a mean value of the network metric of individuals belonging to a given age-sex class after a simulated removal (50 iterations) of randomly selected juveniles. Dotted, dashed and continuous lines represent network metric values of adult females, subadult females and adult males respectively after simulated removals of juveniles. Error bars represent standard errors. Some of the error bars are very narrow and cannot be seen.

**Fig 7:**
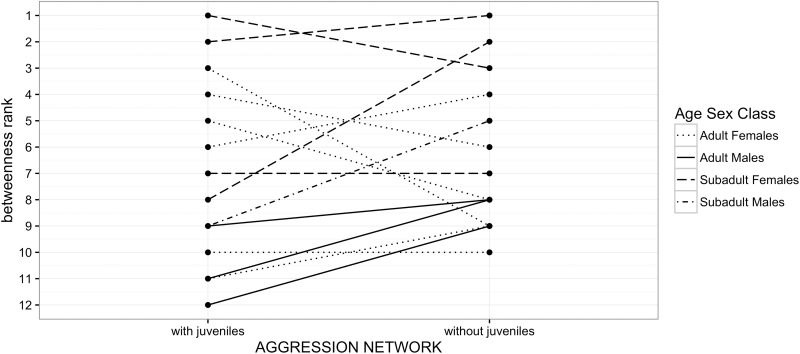
Changes in relative betweenness_Ag_ rank of different age-sex classes resulting from the removal of juveniles in the aggression network.

In line with this we also found that three out of the five most central_Ag_ individuals changed depending on whether or not juveniles were included into the analysis (Fig 7). In addition, three out of the five most central individuals in the network containing juveniles were adults, this changed to four out of the five most central individuals being subadults in the network without juveniles (Fig 7). Interestingly, adult males remained at the bottom of the betweenness_Ag_ rank scale in both scenarios (Fig 7).

Taken together, the removal of juveniles affected males and females as well as adults and subadults differently, with the exact nature of the effect depending on the behaviour used to build the network as well as on the network metric investigated.

## Discussion

Although including juveniles in studies on animal social behaviour might not always be necessary (as it depends on the research question analysed), we show here that care needs to be taken when excluding an entire age-class from social network analyses. In the case of our baboons, juveniles differed from other age-sex classes in terms of most of their social network metrics and including them into the analysis can lead to changes in our interpretation of overall social structure and age-sex specific social roles. Because the effects shown here were highly dependent on the actual network and metric being analysed, it is difficult to generally predict how the exclusion of juveniles will affect social networks. Thus, our study largely serves as a ‘proof of concept’, showing that there is the potential for misinterpretation of social roles and dynamics when an entire age-class is excluded from the study.

### Juvenile sociality and their impact on overall network structure

The omission of juveniles in animal studies is often justified by the assumption that juveniles primarily interact with their mothers or close kin and are thus unlikely to affect the social structure of the group significantly [77–81]. However, studies on a variety of species (e.g. song sparrows,*Melospiza melodia* [82]; feral horses, *Equus caballus* [83]; bottlenose dolphins, *Tursiops sp*. [84]; killer whales, *Orcinus orca* [11]; ring-tailed coatis, *Nasua nasua* [46]; chacma baboons *Papio ursinus* [27–28,42]; bonnet macaques, *Macaca radiata* [26]; rhesus monkeys, *Macaca mulata* [85]; our study) have shown that juveniles do have independent social relationships and as such they should be expected to significantly contribute to overall social network structure.

In our study, some of the effects of juveniles on network structure could be attributed to methodological issues, such as changes in overall density (e.g., changes in clustering coefficient) but this was not the case for all the metrics analysed here, especially not when juveniles were targeted during knock-out simulations (see **S6 Table**). Networks without juveniles were more centralised, less dense and less clustered (grooming network) than networks containing only adults and subadults. This suggests that juveniles either have fewer social partners than adults or that they interact less frequently with other juveniles then with individuals belonging to other age sex classes. Similar binary degree values of juveniles and adults in the two social networks (see **S8 Table)** suggest the latter to be the case.

Overall, we found that the exclusion of individuals had more of an effect on the grooming than the aggression network. This is of particular importance, because grooming behaviour is often used to infer social structure and social bonds in primates (e.g.[3,54]) and other species (e.g. meerkats (*Suricata suricatta)*[86], horses (*Equus ferus)* [55], ring-tailed coatis (*Nasua nasua)* [46]). Our results however suggest that excluding juveniles from these networks may lead to a biased view of the distribution and structure of social bonds, even amongst adults. Interestingly, the aggression network was less affected by the knockout simulations than the grooming network, and effects we found were often not specific to the removal of juveniles, potentially reflecting the response of the network to changes in overall density (but see below). We hypothesize that the exact nature of the effects the exclusion of juveniles will have on network structure is likely to vary between species and will also be dependent on the sex ratio of the juveniles in the group. In our study, most juveniles were males. In baboons, males disperse upon reaching adulthood [31] and as such are not expected to engage in high levels of aggression in their natal troop. In addition, female baboons (and possibly juvenile males until they reach sub-adulthood) inherit their rank position from their mother [42–43,87] and do not usually fight a lot [39]. However, in species where the philopatric sex fights over dominance we would expect to see a much stronger effect of juveniles on the aggression network. In such a species, juveniles may start early to integrate into the social (aggression) network, as has been seen in bottlenose dolphins (*Tursiops sp*.) [84].

Thus, although the exact nature of the effects of the inclusion/exclusion of juveniles will depend on the species and the group sex ratio as well as the behaviour investigated, our results suggest that the exclusion of an entire age-class can have important implications for a variety of social network applications. For example, many studies on the patterns of disease spread in social networks do not include juveniles (e.g., [15–17,88,89]), although age of individuals can be an important factor in terms of infection risk [90,91]. Our results highlight that this practice could be a problem, especially for species in which juveniles are interacting with a substantial proportion of their social group. Moreover, we show that the structure of the grooming network containing a higher proportion of juveniles can be very dissimilar to the one containing only adults. In our example of olive baboons we found that the grooming network including juveniles is actually very similar to random networks which have been shown to have different dynamics in terms of disease (or information) transmission compared to more clustered, ‘small-world’ networks often observed in group living animals [92–94]. Furthermore, the fact that juveniles compared to adult were more central in the grooming network suggests that they may form connections between clusters of individuals that otherwise rarely interact, which, in turn, may potentially facilitate the spread of infectious diseases. As a consequence, the spread of a disease might be over/under-estimated when juveniles are not included into the analysis which, in consequence, can have implications for vaccination programs and disease management.

### Effects of juvenile exclusion on the interpretation of social roles

Age and sex-specific social roles have been described for a large variety of species [11,44,58]. However, we found that the inclusion/exclusion of juveniles can change these interpretations of social roles, as individuals belonging to different age-sex classes can be affected differently by the exclusion of juveniles. For instance, our results show that when juveniles were excluded from the aggression network, subadult and adult females did not differ markedly from each other in terms of network betweenness centrality, while subadults were the age/sex-class with by far the highest betweenness centrality when juveniles were included in the analysis. This may be due to the fact that subadult females in female-bonded cercopithecine primates, such as olive baboons, frequently interact with both juveniles and adults (e.g., chacma baboons [28]; patas monkeys, *Erythrocebus patas* [95]). Moreover, subadult female baboons frequently interact with both, adult males and juveniles while the latter two rarely interact ([27], our study). These differentiated interaction patterns have implications not only for the interpretation of social roles, but also for our understanding of how social cohesion is maintained in networks, as the interactions between juveniles and subadult females affected the overall level of network clustering in the case of our baboons. More generally, it seems that subadults (especially those of the non-dispersing sex) contribute more than previously recognised to the social cohesion of groups as they are likely to interact with both adults and juveniles, thus bridging between these two age classes. This has also been demonstrated for red foxes (*Vulpes vulpes)* [96], spider monkeys (*Ateles geoffroyi*) [97] and yellow-*belied marmots* [98]. Excluding juveniles from social networks in species where subadults have such an integral position in a network will greatly underestimate the importance of this age class for group social network cohesion as well as potentially distorting the real extent of differences in terms of sociality between other age/sex classes.

As predicted, the social network position of males was not strongly affected by the removal of juveniles in the grooming network, due to the fact that adult males rarely groomed juveniles, a finding that corresponds to other studies on primates [99–100]. However, contrary to our predictions, we found that the social network position (especially network clustering) of adult males in the aggression network was strongly affected by the removal of juveniles. The observed increase in clustering coefficient of adult males following the removal of juveniles might be simply due to the fact that adult males rarely interacted aggressively with juveniles (personal observation). Because of the way this metric is calculated, the removal of an age class (here juveniles) is expected to have a stronger influence on mean clustering coefficient of the age-sex class that has few connections with the removed age class (here adult males) than on an age-sex class with whom many connections exist (here adult and sub-adult females). Nevertheless, the rare aggressive interactions that took place between adult males and juveniles had significant implications for the apparent network structure and relative network positions of the other age-sex classes (as indicated by the changes in clustering coefficient) and should as such not be ignored.

It is important to emphasise that the simulated knockouts we conducted here are of course not equivalent to the physical removal of individuals from the group [2,8,21]. Thus, the conclusions about social structure and the influence of juvenile baboons on their social networks *per se* are limited. Instead, the aim of our study was to demonstrate that even though incomplete networks can still provide robust results [12] excluding an entire age or age-sex class from observation or analyses may result in a biased interpretation of social network structure. By comparing network structures resulting from different knock-out simulations we have demonstrated that the (mathematical) inclusion of juveniles into social network analysis affects the conclusions about the relative network position and network connectivity of other individuals in a non-uniform and not always predictable fashion. Moreover, the exclusion of juveniles from social networks is predicted to have very specific effects on the resulting social structure, which are most likely not the same in all species (or even in other baboons). Therefore, more research on a larger number of species along with more simulation studies is needed in order to better understand the effect juveniles have on overall social structure and the extent to which the exclusion of juveniles affects our interpretation of social roles.

In conclusion, using olive baboons as an example, we have provided proof of the concept that the inclusion of juveniles can change the resulting structure of the social networks compared to adult-only networks. This influence is present (but differs in direction and extent) across social behaviours and a range of network metrics. The results of our study thus suggest that a bias in incomplete sampling of social groups, such as omitting juveniles. can lead to an incomplete or distorted representation of age and sex specific social roles in animals.

## Supporting information

S1 Table The percentage of grooming and agonistic interactions in which a given age sex class was observed in relation to the total amount of observed grooming or agonistic interactions. (DOCX)

S2 Table Results of Kendall correlation between specific network metrics used in aim 1 and aim 3 of the study. Significant results are in bold.
(DOCX)

S3 Table Pair-wise Mann Whitney U test results of network density between juveniles and adults. Numbers in the first column represent the number of removed individuals.
(DOCX)

S4 Table Pair-wise Mann Whitney U test results of network clustering between juveniles and adults. Numbers in the first column represent the number of removed individuals.
(DOCX)

S5 Table Pair-wise Mann Whitney U test results of network degree centralisation between juveniles and adults. Numbers in the first column represent the number of removed individuals.
(DOCX)

S6 Table Comparison of observed and simulated network metrics following the removal of all juveniles from the grooming and aggression networks. (DOCX)

S7 Table Comparison of observed and simulated network metrics following the removal of adults from the grooming and aggression networks. (DOCX)

S8 Table Mean values of binary degree for juveniles and adults in the grooming and aggression network. (DOCX)

S1 Text An overview of statistical procedures applied in order to control for the changes in density in knockout simulations. (DOCX)

S2 Text **Fig A.** Density plot of simulated distributions of in degree network metrics of juveniles and adults in the grooming network.
**Fig B.** Density plot of simulated distributions of out degree network of juveniles and adults in the grooming network.
**Fig C.** Density plot of simulated distributions of in strength network of juveniles and adults in the grooming network.
**Fig D.** Density plot of simulated distributions of out strength network of juveniles and adults in the grooming network.
**Fig E.** Density plot of simulated distributions of betweenness centrality of juveniles and adults in the grooming network.
**Fig F.** Density plot of simulated distributions of clustering coefficient of juveniles and adults in the grooming network.
**Fig G.** Density plot of simulated distributions of in degree of juveniles and adults in the aggression network.
**Fig H.** Density plot of simulated distributions of out degree of juveniles and adults in the aggression network.
**Fig I.** Density plot of simulated distributions of in strength of juveniles and adults in the aggression network.
**Fig J.** Density plot of simulated distributions of out strength of juveniles and adults in the aggression network.
**Fig K.** Density plot of simulated distributions of betweenness centrality of juveniles and adults in the aggression network.
**Fig L.** Density plot of simulated distributions of clustering coefficient of juveniles and adults in the aggression network.
(DOCX)
