# Supplementary material for: The effect of excluding juveniles on apparent adult olive baboons (Papio anubis) social networks
Source: PLoS One. 2017 Mar 21;12(3):e0173146. doi: 10.1371/journal.pone.0173146 (PMC5360227; doi:10.1371/journal.pone.0173146)
Supplement: S1 Table — (DOCX) [file pone.0173146.s001.docx]

S1 Table

The percentage of total number of grooming and agonistic interactions in which a given age/sex class was involved.

| Age-sex class | Grooming network | Aggression network |
| --- | --- | --- |
| Juvenile males (N=8) | 43.3% | 37.4% |
| Juvenile females (N=2) | 20.1% | 21.8% |
| Subadult males (N=1) | 3.8% | 11.4% |
| Subadult females (N=4) | 43.9% | 43.5% |
| Adult males (N=3) | 9.2% | 17.9% |
| Adult females (N=7) | 70.1% | 43.5% |
