# Supplementary material for: The effect of excluding juveniles on apparent adult olive baboons (Papio anubis) social networks
Source: PLoS One. 2017 Mar 21;12(3):e0173146. doi: 10.1371/journal.pone.0173146 (PMC5360227; doi:10.1371/journal.pone.0173146)
Supplement: S2 Table — Significant results are in bold. (DOCX) [file pone.0173146.s002.docx]

S2 Table

Results of Kendall rank correlation between specific network metrics used in aim 1 and aim 3 of the study. Significant results of positively correlated metric values are in bold (N=20).

| **Grooming**  **Network** | **Metric** | **In degree** | **Out degree** | **In strength** | **Out strength** | **Betweenness** | **Clustering** |
| --- | --- | --- | --- | --- | --- | --- | --- |
|  | **In degree** |  | tau=0.070  P=0.65 | **tau=0.57**  **P<0.001** | tau=-0.12  P=0.76 | tau=-0.12  P=0.20 | tau=-0.12  P=0.69 |
|  | **Out degree** |  |  | tau=-0.03  P=0.58 | **tau=0.72**  **P<0.001** | **tau=0.60**  **P<0.001** | tau=-0.09  P=0.7 |
|  | **In strength** |  |  |  | tau=-0.15  P=0.83 | tau=0.15  P=0.18 | tau=-0.18  P=0.71 |
|  | **Out strength** |  |  |  |  | **tau=0.56**  **P<0.001** | tau=0.058  P=0.61 |
|  | **Betweenness** |  |  |  |  |  | tau=0.01  P=0.69 |
| **Aggression**  **Network** | **Metric** | **In degree** | **Out degree** | **In strength** | **Out strength** | **Betweenness** | **Clustering** |
|  | **In degree** |  | tau=-0.72  P=<0.001 | **tau=0.75**  **P<0.001** | tau=-0.57  P<0.001 | tau=0.18  P=0.28 | tau=-0.16  P=0.34 |
|  | **Out degree** |  |  | tau=-0.56  P<0.001 | **tau=0.82**  **P<0.001** | tau=-0.14  P=0.41 | tau=0.10  P=0.53 |
|  | **In strength** |  |  |  | tau=-0.45  P=0.005 | tau=0.10  P=0.51 | tau=0  P=1 |
|  | **Out strength** |  |  |  |  | tau=-0.1  P=0.56 | tau=0.13  P=0.46 |
|  | **Betweenness** |  |  |  |  |  | tau=-0.1  P=0.51 |
