# Supplementary material for: The effect of excluding juveniles on apparent adult olive baboons (Papio anubis) social networks
Source: PLoS One. 2017 Mar 21;12(3):e0173146. doi: 10.1371/journal.pone.0173146 (PMC5360227; doi:10.1371/journal.pone.0173146)
Supplement: S3 Table — Numbers in the first column represent the number of removed individuals. (DOCX) [file pone.0173146.s003.docx]

S3 Table

Pair-wise Mann Whitney U test results of network density between juveniles and adults. Numbers in the first column represent the number of removed individuals. Significant results are indicated in bold.

| Number of individuals | Grooming Network | Agonistic Network |
| --- | --- | --- |
| 1 | U = 51.5 p = 0.91 | U = 48.5 p = 0.9 |
| 2 | U = 1040.5 p = 0.82 | U = 1021.5 p = 0.941 |
| 3 | U = 1040.5 p = 0.82 | U = 7650 p = 0.4 |
| 4 | U = 23701 p = 0.18 | **U = 24785 p = 0.03** |
| 5 | **U = 35154.5 p = 0.04** | **U = 38327.5 p<0.001** |
| 6 | **U = 25317 p = 0.008** | **U = 29106.5 p<0.001** |
| 7 | **U = 8710 p = 0.005** | **U = 10670 p<0.001** |
| 8 | **U = 1325 p = 0.01** | **U = 1707.5 p<0.001** |
| 9 | U = 75 p = 0.06 | **U = 98.5 p<0.001** |
