# Supplementary material for: The effect of excluding juveniles on apparent adult olive baboons (Papio anubis) social networks
Source: PLoS One. 2017 Mar 21;12(3):e0173146. doi: 10.1371/journal.pone.0173146 (PMC5360227; doi:10.1371/journal.pone.0173146)
Supplement: S4 Table — Numbers in the first column represent the number of removed individuals. (DOCX) [file pone.0173146.s004.docx]

S4 Table

Pair-wise Mann Whitney U test results of network clustering between juveniles and adults. Numbers in the first column represent the number of removed individuals. Significant results are indicated in bold.

| Number of individuals | Grooming Network | Agonistic Network | |
| --- | --- | --- | --- |
| 1 | W = 65 p = 0.28 | W = 44 p = 0.68 |  |
| 2 | **W = 1481 p<0.001** | W = 833 p = 0.15 |  |
| 3 | **W = 11596 p<0.001** | **W = 5606 p = 0.003** |  |
| 4 | **W = 37896 p<0.001** | **W = 16988 p<0.001** |  |
| 5 | **W = 57402 p<0.001** | **W = 24638 p<0.001** |  |
| 6 | **W = 41616 p<0.001** | **W = 17414 p<0.001** |  |
| 7 | **W = 14036 p<0.001** | **W = 5859 p = 0.01** |  |
| 8 | **W = 2012 p<0.001** | W = 829 p = 0.14 |  |
| 9 | **W = 100 p<0.001** | W = 36 p = 0.31 |  |
