# Supplementary material for: The effect of excluding juveniles on apparent adult olive baboons (Papio anubis) social networks
Source: PLoS One. 2017 Mar 21;12(3):e0173146. doi: 10.1371/journal.pone.0173146 (PMC5360227; doi:10.1371/journal.pone.0173146)
Supplement: S5 Table — Numbers in the first column represent the number of removed individuals. (DOCX) [file pone.0173146.s005.docx]

S5 Table

Pair-wise Mann Whitney U test results of network centralisation between juveniles and adults. Numbers in the first column represent the number of removed individuals. Significant results are indicated in bold.

| Number of  individuals | Grooming Network | Agonistic Network |
| --- | --- | --- |
| 1 | U = 58 p = 0.54 | U = 64.5 p = 0.27 |
| 2 | **U = 1258 p = 0.04** | **U = 1341.5 p = 0.008** |
| 3 | **U = 11359 p<0.001** | **U = 9797.5 p<0.001** |
| 4 | **U = 38075 p<0.001** | **U = 31101 p<0.001** |
| 5 | **U = 58263 p<0.001** | **U = 46144.5 p<0.001** |
| 6 | **U = 42378.5 p<0.001** | **U = 32795 p<0.001** |
| 7 | **U = 14256.5 p<0.001** | **U = 10776.5 p<0.001** |
| 8 | **U = 2025 p<0.001** | **U = 1526 p<0.001** |
| 9 | **U = 100 p<0.001** | **U = 78.5 p = 0.03** |
