# Supplementary material for: The effect of excluding juveniles on apparent adult olive baboons (Papio anubis) social networks
Source: PLoS One. 2017 Mar 21;12(3):e0173146. doi: 10.1371/journal.pone.0173146 (PMC5360227; doi:10.1371/journal.pone.0173146)
Supplement: S6 Table — (DOCX) [file pone.0173146.s006.docx]

S6 Table

Comparison of observed and simulated network metrics following the removal of all juveniles (N=10) from the grooming and aggression networks. Simulated values are mean values (± standard deviation) of 1000 random networks exhibiting the same density and the same range of weights as the original networks after the removal of juveniles. Network values differing more than 2SD from the corresponding values of the random networks are in bold.

| Network metric | Network type | Grooming network | Aggression network |
| --- | --- | --- | --- |
| Clustering  coefficient | Observed | 0.479 | **0.621** |
|  | Random | 0.408 ±0.072 | 0.524± 0.047 |
| Network degree  centralisation | Observed | **0.291** | 0.127 |
|  | Random | 0.145±0.042 | 0.159±0.048 |
