# Supplementary material for: The effect of excluding juveniles on apparent adult olive baboons (Papio anubis) social networks
Source: PLoS One. 2017 Mar 21;12(3):e0173146. doi: 10.1371/journal.pone.0173146 (PMC5360227; doi:10.1371/journal.pone.0173146)
Supplement: S8 Table — (DOCX) [file pone.0173146.s008.docx]

S8 Table

Mean values ± SD of binary degree for juveniles (n=10) and adults (n=10) in the grooming and aggression network.

|  | Grooming | Aggression |
| --- | --- | --- |
| Juveniles | 7.6±3.13 | 10.7±2.98 |
| Adults | 8.2±3.52 | 10.7±1.70 |
