# Supplementary material for: The effect of excluding juveniles on apparent adult olive baboons (Papio anubis) social networks
Source: PLoS One. 2017 Mar 21;12(3):e0173146. doi: 10.1371/journal.pone.0173146 (PMC5360227; doi:10.1371/journal.pone.0173146)
Supplement: S1 Text — (DOCX) [file pone.0173146.s009.docx]

S1 Text

*Controlling for the effect of density on the network metrics*

Other network metrics, such as clustering coefficients, have also been shown to be sensitive to changes in network density (Brent et al. 2013, Croft et al. 2008). To assess if changes in density could explain the changes in network measures following the removal of individuals, we simulated 1000 random networks with the same density and identical range of weights as the ‘observed’ networks following the removal of 10 adults or juveniles. For these simulated networks we calculated the two global networks metrics (clustering and degree centralisation dropping density, as this was a fixed parameter in the simulation) and compared the resulting values to our ‘observed’ values. A significant difference between the simulated and ‘observed’ means would suggest that the observed changes in network structure following our knock-out simulations are not simply due to changes in density but instead are specific to the removal of individuals. We considered differences to be significant when observed values were outside the mean ± 2 standard deviations range of the values from the randomly generated networks.

As the removal of juveniles (our main focus) changed network density significantly for both networks (Table 3), we conducted a control experiment to assess if the observed changes in network metrics following our knock-out simulations could be solely attributed to changes in density. We found that in 50% of the comparisons (two out of four), the observed network metrics following juvenile removals were significantly (i.e., more than 2 SD) different from those expected from 1000 randomised networks of the same density (Table 4S). In contrast, following the removal of adults only 25% (one out of four) of the observed network metrics differed significantly from what would be expected from random networks with the same density and range of weights: clustering in the aggression network (Table 5S). This suggests that networks resulting from the removal of adults (i.e. with a higher proportion of juveniles), are more similar to random networks whereas networks with a higher proportion of adults (following the removal of juveniles) are different from random and the observed effects (see Fig. 2 and 3 in the main text) are most likely not a consequence of changes in density suggesting that adults to a greater extent maintain non-random network structure compared to juveniles.
