# Supplementary material for: The effect of excluding juveniles on apparent adult olive baboons (Papio anubis) social networks
Source: PLoS One. 2017 Mar 21;12(3):e0173146. doi: 10.1371/journal.pone.0173146 (PMC5360227; doi:10.1371/journal.pone.0173146)
Supplement: S2 Text — Fig A. Density plot of simulated distributions of in degree network metrics of juveniles and adults in the grooming network. Fig B. Density plot of simulated distributions of out degree network of juveniles and adults in the grooming network. Fig C. Density plot of simulated distributions of in strength network of juveniles and adults in the grooming network. Fig D. Density plot of simulated distributions of out strength network of juveniles and adults in the grooming network. Fig E. Density plot of simulated distributions of betweenness centrality of juveniles and adults in the grooming network. Fig F. Density plot of simulated distributions of clustering coefficient of juveniles and adults in the grooming network. Fig G. Density plot of simulated distributions of in degree of juveniles and adults in the aggression network. Fig H. Density plot of simulated distributions of out degree of juveniles and adults in the aggression network. Fig I. Density plot of simulated distributions of in strength of juveniles and adults in the aggression network. Fig J. Density plot of simulated distributions of out strength of juveniles and adults in the aggression network. Fig K. Density plot of simulated distributions of betweenness centrality of juveniles and adults in the aggression network. Fig L. Density plot of simulated distributions of clustering coefficient of juveniles and adults in the aggression network. (DOCX) [file pone.0173146.s010.docx]

S2 Text

Fig A. Density plots of simulated distributions (N=252) of in degree values of adults and juveniles in the grooming network.

Fig B. Density plots of simulated distributions (N=252) of out degree values of adults and juveniles in the grooming network.

Fig C. Density plots of simulated distributions (N=252) of in strength values of adults and juveniles in the grooming network.

Fig D. Density plots of simulated distributions (N=252) of out strength values of adults and juveniles in the grooming network.

Fig E. Density plots of simulated distributions (N=252) of betweenness centrality of adults and juveniles in the grooming network.

Fig F. Density plots of simulated distributions (N=252) of clustering coefficient of adults and juveniles in the grooming network.

Fig G. Density plots of simulated distributions (N=252) of in degree values of adults and juveniles in the aggression network.

Fig H. Density plots of simulated distributions (N=252) of out degree values of adults and juveniles in the aggression network.

Fig I. Density plots of simulated distributions (N=252) of in strength values of adults and juveniles in the aggression network.

Fig J. Density plots of simulated distributions (N=252) of out strength values of adults and juveniles in the aggression network.

Fig K. Density plots of simulated distributions (N=252) of betweenness centrality of adults and juveniles in the aggression network.

Fig L. Density plots of simulated distributions (N=252) of clustering coefficient of adults and juveniles in the aggression network.
